# Burden and Economic Impact of Respiratory Viral Infections in Adults Aged 60 and Older: A Focus on RSV

**DOI:** 10.3390/diseases13020035

**Published:** 2025-01-28

**Authors:** Adrián Peláez, Sara Jimeno Ruiz, Mercedes Villarreal, Manuel Gil, Inés Gutiérrez, Marta Sanz, Silvina Natalini Martínez

**Affiliations:** 1 Facultad de Ciencias de la Salud-HM Hospitales, Universidad Camilo José Cela, 28692 Madrid, Spain; 2 Centro de Investigación en Red de Enfermedades Respiratorias (CIBERES), Instituto de Salud Carlos III (ISCIII), 28029 Madrid, Spain; 3 Departamento de Pediatría, Hospital HM Puerta del Sur, HM Hospitales, 28938 Madrid, Spain; sarajimeno@hotmail.com (S.J.R.); slnatalini@hmhospitales.com (S.N.M.); 4 Unidad de Vacunas, HM Hospitales, 28938 Madrid, Spain; 5 Departamento de Medicina Interna, Hospital HM Torrelodones, Hospital HM, 28250 Madrid, Spain; mvillarreal@hmhospitales.com (M.V.); mgil@hmhospitales.com (M.G.); igutierrezg@hmhospitales.com (I.G.); martasanzrubio@empleado.hmhospitales.com (M.S.)

**Keywords:** respiratory syncytial virus, RSV, older adults, economic burden, comorbidities, vaccination, acute respiratory infections

## Abstract

**Background/Objectives:** Respiratory syncytial virus (RSV) represents a significant cause of acute respiratory infections (ARIs) in adults aged 60 years and older, often leading to severe clinical outcomes and high healthcare costs. This study aimed to evaluate the clinical and economic burden of RSV compared to other ARIs, focusing on specific age groups, comorbidities, and demographic factors. **Methods:** A retrospective observational study was conducted using the electronic medical records of adults aged ≥60 years hospitalized for ARIs, including RSV, in Spain. Direct costs related to hospitalizations, intensive care unit (ICU) admissions, and treatments were analyzed. The study also assessed demographic, clinical, and comorbidity-related factors influencing the economic burden. **Results:** RSV infections resulted in significantly higher direct costs compared to other ARIs, particularly in patients aged 70–80 years. Comorbidities such as asthma and smoking history were associated with increased costs in RSV cases. Although ICU costs were comparable between groups, hospitalizations for RSV required longer stays and more intensive treatments, amplifying the overall economic burden. Differences in costs by age and sex highlighted the need for tailored clinical management strategies. **Conclusions:** RSV poses a substantial economic and clinical burden on adults aged 60 years and older, particularly in those with comorbidities. Preventive measures, such as vaccination, could reduce healthcare costs and improve outcomes in this vulnerable population. These findings support the inclusion of RSV vaccines in immunization programs, especially in aging populations like Spain, to alleviate healthcare pressures during peak respiratory disease seasons.

## 1. Introduction

Respiratory infections are among the leading causes of morbidity and mortality globally, particularly in older adults, with significant repercussions for public health systems and healthcare costs [1,2,3]. In 2016, respiratory infections accounted for approximately 2.38 million deaths worldwide, ranking as the sixth leading cause of mortality overall and the leading cause of death in children under five years old [4,5]. The aging population, especially in high-income countries, further exacerbates the impact of these infections, as older adults often face higher hospitalization costs and a greater burden of comorbidities [6,7]. Among respiratory pathogens, respiratory syncytial virus (RSV) has emerged as a significant cause of severe respiratory disease in elderly individuals, particularly those with chronic conditions or residing in nursing homes, where transmission rates are high [8,9,10]. RSV is estimated to cause over 470,000 hospitalizations and 33,000 deaths annually in industrialized countries, underscoring the pressing need for effective prevention, early diagnosis, and treatment strategies [3,11,12,13,14,15,16].

Advancements in diagnostic techniques have enhanced our understanding of the role of viral agents, including RSV, in acute respiratory diseases in older adults over the past two decades [8,9,17,18]. However, diagnostic challenges persist due to difficulties in clinical recognition, insufficient availability of routine tests, and limitations in detecting low viral loads, which contribute to the underdiagnosis of RSV infection [12,13,14,15]. RSV has a significant economic impact on healthcare systems, with medical care for older adults generating billions of dollars in direct and indirect costs annually [19,20,21,22,23,24,25,26]. In Spain, hospitalizations related to RSV represent a considerable expense for the National Health System, further emphasizing the need for measures to mitigate this economic burden and optimize healthcare resources [27,28].

Although previous studies have highlighted the clinical and economic burden of RSV, most focus on pediatric populations or specific high-risk groups [25,26,27,28,29,30], leaving a gap in comprehensive analyses of its impact on older adults. This study adds to the existing literature by providing a detailed evaluation of the economic burden of RSV in adults aged 60 and over across different regions of Spain. By integrating epidemiological and healthcare cost data, it offers a nuanced understanding of the direct and indirect costs associated with RSV hospitalizations in this demographic.

By prioritizing prevention, early diagnosis, and improved vaccination strategies, healthcare systems can reduce the burden of RSV and other viral respiratory infections in older adults, especially those with comorbidities [15,31,32]. This study aims to evaluate the economic impact of RSV in adults aged 60 and over across different regions of Spain, providing evidence to guide public health decision-making and inform the design of effective intervention strategies.

## 2. Materials and Methods

A retrospective, multicenter observational study was conducted on older adult patients (≥60 years) diagnosed with RSV infection, confirmed by PCR or antigen testing, between 1 October 2023 and 31 March 2024. The decision to focus on adults aged 60 and above stems from the extensively documented heightened susceptibility of this age group to respiratory infections, especially RSV, along with the greater strain these cases place on healthcare systems [3,20,21,24]. The study included anonymized secondary data extracted from the electronic health records (EHR) of patients admitted to 18 university hospitals within the HM Hospitales network, located across Spain in Madrid, León, Cataluña, Galicia, and Andalucía. Inclusion criteria required patients to: (1) be aged 60 years or older at the time of admission; (2) have a confirmed diagnosis of RSV infection through PCR or antigen testing during the study period (1 October 2023 to 31 March 2024); and (3) require hospitalization primarily for respiratory symptoms attributed to RSV, as documented in their medical records. Patients were excluded if they had incomplete or missing key data in their EHR, including diagnostic results, clinical details, or discharge outcomes. Similarly, patients hospitalized primarily for conditions unrelated to respiratory infections (e.g., trauma, cardiovascular events, or elective procedures) were excluded.

Sociodemographic and clinical variables were collected, along with results from antigen and PCR tests for respiratory viruses. Data on pharmacological treatments, oxygen therapy requirements, and the need for invasive or non-invasive ventilation were also gathered. Information was extracted from the Doctoris EHR system, ensuring all patient identifiers were anonymized to protect confidentiality. A detailed breakdown of these cost estimates can be found in Table S1 of the supplementary materials.

A preliminary descriptive analysis of patient characteristics was performed. Measures of central tendency and dispersion were calculated for quantitative variables, while counts and percentages were used for qualitative variables. Differences between groups were assessed using normality tests (Shapiro–Wilk, Kolmogorov–Smirnov) and homogeneity tests (Levene test). Based on these results, parametric tests (ANOVA, Student’s t-test) or non-parametric tests (Kruskal–Wallis, Wilcoxon rank-sum) were applied. For qualitative variables, χ² or Fisher’s exact tests were used.

To assess the impact of RSV vaccination, an efficacy value supported by previous studies was used. These projections indicated that vaccination could reduce severe RSV cases by 94.1% in the first year and 82.7% in the second year, whereas non-severe cases could decrease by 82.6% and 74.5%, respectively [33]. This efficacy value was used to estimate potential reductions in hospitalizations and other RSV-related outcomes in the study population.

To identify and quantify comorbidities with significant impacts on healthcare costs, mixed-effects models were developed. These included a simple linear model and mixed-effects models with random intercept, random slope, and both random intercept and slope. Model comparisons were conducted using goodness-of-fit metrics, such as Akaike Information Criterion (AIC) and Bayesian Information Criterion (BIC), applied to the logarithm of total cost per patient. A random sample of patients with high-cost comorbidities was used for case reduction estimations. Data analysis and visualization were conducted using RStudio.

## 3. Results

### 3.1. Sociodemographic, Clinical, and Healthcare Cost Comparison Between Respiratory Diseases and RSV

The total costs associated with episodes of acute respiratory infection (ARI) and respiratory syncytial virus (RSV) cases were compared across various sociodemographic and clinical aspects. As shown in Table 1, the data indicate that the average total cost for ARI episodes is €3329.17 (±€7175.97), while for RSV episodes it is €5196.96 (±€10,019.56), with a statistically significant difference (*p* < 0.001). The standard deviation reflects the variability in costs within each group, influenced by factors such as the severity of cases, comorbidities, or required treatments.

In terms of costs related to readmissions, patients with RSV incur significantly higher costs compared to ARI patients. For a single admission, the average cost in RSV cases is €8075.52 (±€13,940.15), compared to €3588.41 (±€7502.95) in ARIs (*p* < 0.001). For two readmissions, the average costs are €3416.96 (±€4540.46) for RSV and €3017.18 (±€6875.64) for ARIs (*p* < 0.001). For more than two readmissions, costs are €2721.14 (±€4156.28) for RSV versus €3095.21 (±€6445.11) for ARIs (*p* < 0.001).

Regarding age groups, patients aged 70–80 years with RSV incur significantly higher costs (€7800.36 ± €17,474.31) compared to ARI patients (€3370.93 ± €8510.16) (*p* < 0.001). However, for patients aged 80–90 years and over 90 years, the cost differences between RSV and ARIs are not statistically significant (*p* = 0.140 and *p* = 0.170, respectively).

Total costs associated with ICU hospitalization show that the average cost is €21,006.79 (±€22,851.57) for ARIs and €21,828.70 (±€33,100.00) for RSV, with no significant differences between the two groups (*p* = 0.773).

Regarding comorbidities, patients with RSV have higher costs compared to ARI patients for asthma (€5793.11 ± €4880.71 vs. €3169.43 ± €4064.99, *p* = 0.035) and for former smokers (€5736.77 ± €6893.49 vs. €4408.68 ± €6855.51, *p* = 0.021). However, the cost differences for other comorbidities analyzed, such as diabetes, hypertension, and COPD, are not statistically significant.

### 3.2. Total Costs and RSV Case Reduction

**Figure 1:**
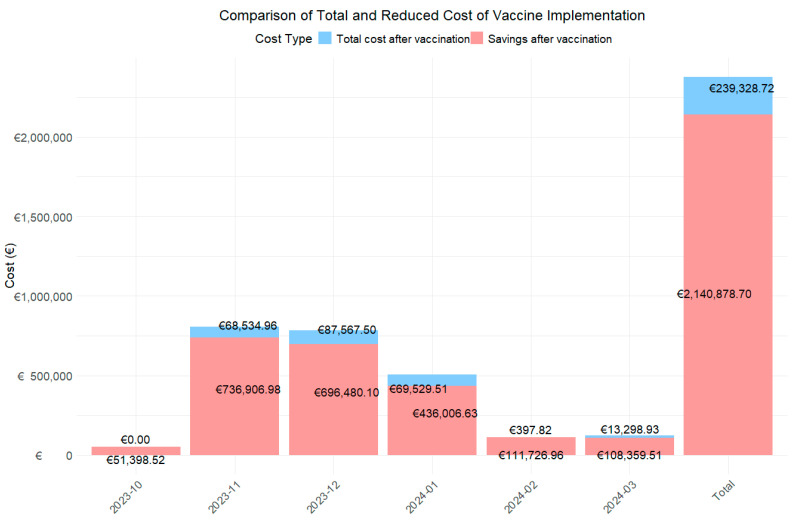
Bar chart to assess total expenditure and savings from vaccination using first and second year cost.

A cost analysis of RSV cases was conducted from October 2023 to March 2024 (Table 2; Figure 1) to assess the economic impact of introducing the RSV vaccine. The study projected that the vaccine would reduce severe RSV cases by 94.1% in the first year and by 82.7% in the second year of protection. In non-severe cases, reductions were expected to be 82.6% in the first year and 74.5% in the second year.

The data show a notable reduction in monthly and total costs. The global analysis for the period from October 2022 to March 2023 revealed that the total cost associated with RSV cases without the vaccine was €1,190,103.71 from a total of 229 RSV cases. With the introduction of the hypothetical vaccine, cases would be reduced to 21 in the first year and 47 in the second year, with an associated cost of €89,448.42 and €216,320.30, representing an overall decrease of 92.5% and 81.8%.

When analyzed by month, the three months with the highest costs during the RSV season were November (€402,720.97), December (€392,023.80), and January (€252,768.07), coinciding with the epidemic peak. During these months, 46 cases, 77 cases, and 70 RSV cases were reported, respectively. With the introduction of the hypothetical vaccine, it is projected that RSV cases will be reduced to 13, 22, and, 23 cases in two years, respectively. This would result in total costs of €13,991.61 and €54,543.35, €13,991.61 and €73,575.89, and €28,528.47 and €41,001.04, representing cost reductions of 96.5% and 86.5%, 96.4% and 81.2%, and 88.7% and 83.8% in each of these months, respectively.

When analyzing the costs associated with the use of the vaccine over two years (Figure 1), the total cost after its implementation is estimated at €239,328.72, resulting in savings of €2,140,878.70 over this period. Furthermore, during the peak epidemic months, the estimated savings would amount to €68,534.96, €87,567.50, and €69,529.51, respectively.

Finally, cost reductions in percentage of change modified notably between the first and second projection years, reflecting the vaccine’s declining effectiveness over time. During peak epidemic months, reductions were highest in the first year: 96.5% in November, 96.4% in December, 88.7% in January, 100% in February, and 99.0% in March, with the largest difference of 19.9 percentage points observed in March.

### 3.3. Total Costs and RSV Case Reduction by Age Range

**Figure 2:**
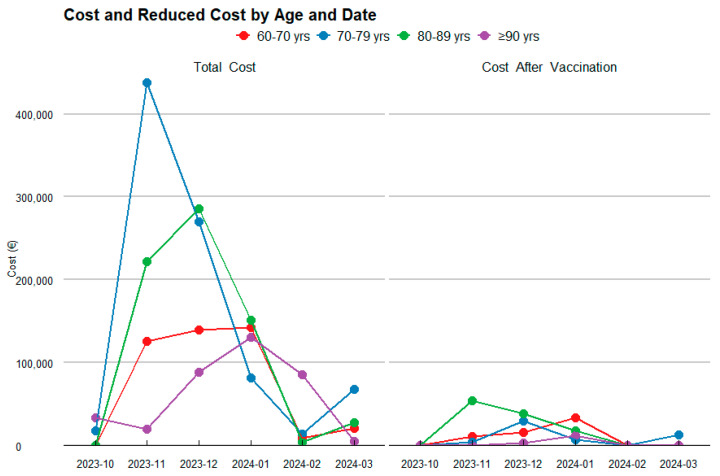
Temporal Evolution of Costs and their Reduction by Age Category.

To more thoroughly assess the financial impact of the RSV vaccine, a detailed analysis was conducted across different age groups, considering both the estimated number of patients and the total healthcare costs before and after vaccination (Table 3; Figure 2).

**Figure 3:**
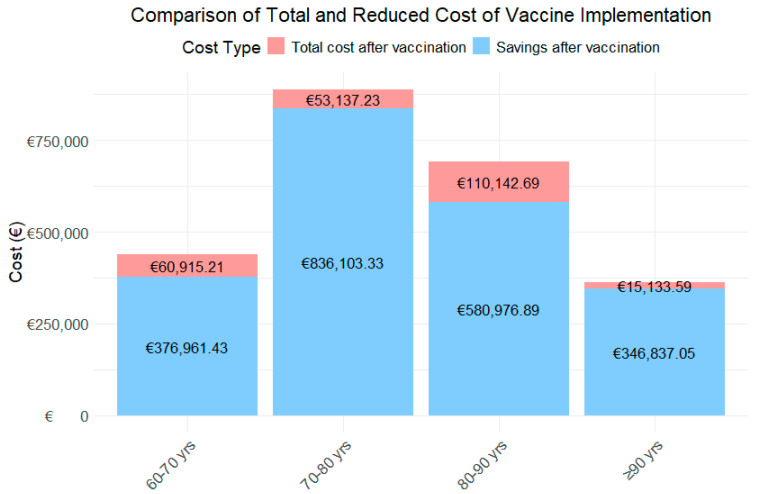
Costs and their Reduction by Age Category.

For the 60–70-year-old age group, the analysis projects that the vaccine will result in a substantial reduction in RSV cases, as shown in Figure 3, decreasing from 55 to 18 cases. This reduction leads to an 88.1% decrease in the first year, and a 84.1% decrease in the second year in healthcare costs, dropping from an estimated total of €218,938.32 to €26,097.08 and €34,818.13 in the first and second year, respectively.

In the 70–80-year-old group, the vaccine is projected to reduce RSV cases from 57 to 16, reflecting a 99.2% decrease in the first year, and a 88.9% decrease in the second year in healthcare costs, with savings of €444,620.28, reducing the total to €35,753.37 in the first year and €49,563.86 in the second year.

For the 80–90-year-old group, a significant reduction in RSV cases is observed, from 80 to 25, resulting in a 93.0% decrease in the first year, and a 75.1% decrease in the second year. This reduction leads to savings of €345,559.79, bringing the total cost down to €24,262.42 and €85,880.27.

Finally, in the 90+ years age group, the vaccine has a notable impact, reducing RSV cases from 37 to 9 and cutting the associated healthcare costs by 98.2% in the first year and 93.4% in the second year. The total spending decreases from €180,985.32 to €3175.55 and €11,958.04.

When analyzing the total costs by age group over the two-year period (Figure 3), the age group with the highest expenditure is those aged 80–90 years, followed by 60–70 years, 70–80 years, and finally those aged ≥90 years. In contrast, the age group achieving the greatest savings is 70–80 years, followed by 80–90 years, and the 90+ years age group.

### 3.4. Total Costs and RSV Case Reduction Due to the Most Important Comorbidities

In order to identify and quantify comorbidities that have a significant impact on total healthcare costs in a patient population, several mixed-effects models with varying complexity were developed. First, a simple linear model was created, along with a mixed-effects linear model with random intercept (patient identifier as a random effect to model individual variability), random slope (age as a random effect to capture heterogeneity between patients), and both random intercept and slope (identifier and age as random effects). The four models were compared to fit the logarithm of the total cost per patient. The simple linear model had the lowest AIC (6754.621) and BIC (6849.309), indicating the best balance between fit and simplicity. The mixed models with random intercept (AIC: 6800.141), random slope (AIC: 6808.580), and both random intercept and slope (AIC: 6812.580) did not show significant improvements in fit, according to the likelihood ratio test. Although the mixed models capture individual variability, the simple linear model proved to be the most suitable due to its simplicity and robust fit.

The results from the simple linear model (Table S2) revealed significant associations between various comorbidities and healthcare costs. First, all evaluated comorbidities, except for obesity (coefficient = 0.051, *p* = 0.639), alcoholism (coefficient = 0.105, *p* = 0.518), and being a former smoker (coefficient = 0.093, *p* = 0.255), showed significant increases in total costs. Among the comorbidities that contributed most to the increase in healthcare spending, pneumonia and bronchitis were found, with coefficients of 1.942 and 1.783, respectively (*p*-values < 0.001). Additionally, acute infections showed higher increases in total cost (coefficients 1.412, *p*-value < 0.001), compared to the other significant variables. Using the results shown in Table 4, we evaluated the total cost and potential reductions in healthcare expenditures that an RSV vaccine could bring for patients with comorbidities significantly impacting healthcare costs.

## 4. Discussion

The findings of this study highlight the significant clinical and economic burden associated with RSV infections in adults aged 60 years and older. Compared to other ARIs, episodes caused by RSV result in substantially higher costs, particularly in specific age groups, such as patients aged 70–80 years. This underscores the importance of prioritizing prevention and control strategies for this vulnerable group, given their increased susceptibility to severe complications and associated hospital costs [3,19,21,22]. Older adults, particularly those with comorbid conditions such as heart disease, diabetes, and chronic respiratory diseases, are at greater risk of severe RSV outcomes [21,22]. This demographic also tends to have longer hospital stays and a higher likelihood of requiring intensive care, contributing to the substantial financial burden observed in our study.

The analysis of direct costs reveals that RSV-linked hospitalizations and readmissions represent a substantial economic burden on healthcare systems, significantly exceeding the costs observed for other ARIs. This increase is due to the need for intensive care, prolonged hospital stays, and more complex treatments, especially in patients with comorbidities. The high costs are exacerbated by the need for advanced supportive care such as non-invasive and invasive mechanical ventilation, both of which are associated with prolonged ICU stays and resource-intensive care protocols. The significant financial impact observed highlights the need for healthcare systems to better allocate resources and consider cost-effective prevention strategies to mitigate these costs [27]. Furthermore, the significant differences observed among sex and age groups suggest that clinical management should consider demographic and clinical characteristics to optimize resource utilization. Tailoring treatment and prevention strategies based on age, sex, and comorbidities may enhance care delivery and reduce unnecessary resource utilization, ultimately leading to cost savings for the healthcare system.

Regarding comorbidities, conditions such as asthma, COPD, or a history of smoking are associated with higher costs in patients with RSV compared to those with other ARIs. This finding highlights the importance of identifying and managing risk factors that may exacerbate the clinical and economic impact of RSV [20,23]. Patients with these comorbid conditions require more intensive management and are more likely to experience complications, necessitating longer hospital stays and more expensive treatments. This highlights the potential for targeted interventions to mitigate the impact of RSV in these high-risk individuals, such as preemptive care measures, vaccination, and lifestyle modifications aimed at reducing exacerbations. Although no significant differences in the costs of ICU admissions were observed between groups, the overall ICU burden on healthcare systems remains notable and deserves attention. Future research should explore the direct and indirect consequences of ICU utilization and assess how optimizing ICU capacity could lead to more cost-effective management of RSV cases.

From a public health perspective, these results underscore the value of preventive strategies, particularly RSV vaccination. Implementing immunization programs targeting older adults could yield benefits not only in health outcomes but also in economic savings [15,31,32]. Vaccination could reduce immediate costs related to hospital care and complications, while improving the quality of life of patients with comorbidities [6,16,34]. It should be noted that the effect of vaccination in these patients is especially valuable considering that the duration of protection offered by the vaccine extends beyond a single season, as demonstrated by several recent publications [33,34]. The potential for long-lasting immunity could reduce the need for repeated treatments and hospitalizations, further alleviating the burden on healthcare systems. Moreover, the cost-effectiveness of vaccination programs has been demonstrated in other studies, where reductions in RSV-related hospitalizations, ICU admissions, and overall healthcare costs were observed following the introduction of vaccination programs [33,34]. By investing in vaccines with demonstrated long-term efficacy, healthcare systems can potentially achieve long-term savings while simultaneously improving patient outcomes.

In addition, these vaccination programs for the most vulnerable population groups could reduce pressure on healthcare systems during peak respiratory disease seasons, optimizing resource allocation and reducing morbidity and mortality rates. The ability to prevent or reduce the severity of RSV infections in high-risk patients would also free up hospital resources, potentially lowering the strain on emergency departments and ICU capacity. This, in turn, could allow for better care of other medical conditions, enhancing the overall functioning of healthcare systems, particularly during peak seasons for respiratory diseases.

While our study provides important insights into the clinical and economic burden of RSV in older adults, several limitations should be acknowledged. The retrospective nature of the study and reliance on electronic medical records could introduce biases in data collection, such as missing or incomplete data, which may have led to the exclusion of certain patients or inaccuracies in cost estimation. Additionally, the absence of data on indirect costs, such as productivity loss due to illness or the financial burden on caregivers, represents a gap in our analysis. Indirect costs are particularly important in the elderly population, where caregivers often play a critical role in managing daily activities and recovery. Future studies should address these gaps to provide a more comprehensive assessment of the economic and social impact of RSV in this population. Moreover, the impact of vaccination on indirect costs would be valuable, as it could further highlight the broader economic benefits of RSV prevention.

Another important limitation is the generalizability of our findings beyond the Spanish context. While the study provides valuable insights into the situation in Spain, healthcare systems, demographics, and healthcare policies can vary significantly across different countries. Differences in healthcare infrastructure, vaccination coverage, and the prevalence of comorbidities could influence the clinical and economic burden of RSV, as well as the effectiveness and cost-effectiveness of vaccination programs. For example, when comparing our findings with international studies, we observe that the costs associated with RSV infections in older adults in Spain are in line with those of other European countries, although there are some important differences [35]. These comparisons highlight how variations in healthcare infrastructure, immunization coverage, and comorbidity management can influence both the clinical outcomes and economic burden of RSV infections. Therefore, while our findings are highly relevant for Spain, further research is needed to assess the applicability of these results in other regions, particularly in countries with different healthcare systems or demographic profiles. Comparative studies across different countries or regions could help with better understanding how to optimize RSV vaccination strategies and resource allocation globally.

## 5. Conclusions

Our study underscores the urgent need to prioritize preventive measures, particularly vaccination, to alleviate both the clinical and economic impact of RSV in older adults. By demonstrating the potential benefits of immunization, our study provides compelling evidence to support the implementation of national routine immunization programs, especially in aging populations, such as Spain. This would not only improve health outcomes but also reduce the pressure on healthcare systems, ultimately contributing to better resource management and quality of life for older adults.

## Figures and Tables

**Table 1:** Total cost for each sociodemographic and clinical characteristics for ARI in comparison with RSV cases.

Characteristics	Total ARI (*n*1 = 1952)	Total RSV Cases (*n*2 = 229)	*p*-Value
**No. of readmissions**			
1 admission	3588.41 (±7502.95)	8075.52 (±13,940.15)	**<0.001**
2 admissions	3017.18 (±6875.64)	3416.96 (±4540.46)	**<0.001**
>2 admissions	3095.21 (±6445.11)	2721.14 (±4156.28)	**<0.001**
**Sex [Female]**			
Male	3751.76 (±7889.96)	6884.91 (±14,269.76)	**<0.001**
Female	2972.09 (±6494.35)	4000.28 (±4970.47)	**<0.001**
**Age group**			
60–70 years old	2975.96 (±7849.17)	3980.7 (±4237.2)	**<0.001**
70–80 years old	3370.93 (±8510.16)	7800.36 (±17,474.31)	**<0.001**
80–90 years old	3421.95 (±5586.12)	4319.5 (±6285.38)	0.140
+90 years old	3716.71 (±4752.43)	4891.5 (±5692.02)	0.170
**Days of hospitalization**			
<10 days	3606.67 (±1806.36)	3545.38 (±1956.24)	0.981
10–30 days	11,695 (±6052.03)	9830.16 (±5561.21)	0.117
>30 days	43,620.57 (±22,863.61)	52,628.73 (±39,088.03)	0.813
**UCI**	21,006.79 (±22,851.57)	21,828.7 (±33,100)	0.773
**Days of ICU hospitalization**			
<10 days	18,070.09 (±20,022.44)	21,828.7 (±33,100)	0.975
10–30 days	45,105.77 (±13,636.62)	NA (±NA)	NA
>30 days	100,813.28 (±NA)	NA (±NA)	NA
**Exitus**	12,661.34 (±13,701.38)	8042.49 (±6768.67)	0.454
**Neoplasia**	4908.82 (±9731.23)	7449.31 (±15,737.77)	
**Asthma**	3169.43 (±4064.99)	5793.11 (±4880.71)	**0.035**
**Current smoker**	5099.17 (±8507.84)	5109.19 (±6316.44)	0.502
**Former smoker**	4408.68 (±6855.51)	5736.77 (±6893.49)	**0.021**
**Alcoholism**	7124.05 (±16,753.31)	21,007.81 (±43,829.38)	0.156
**Diabetes**	4454.19 (±7891.93)	5031.63 (±5194.22)	0.078
**Obesity**	3872.33 (±5250.17)	3983.81 (±3732.9)	0.422
**Hypertension**	3969.77 (±7317.09)	5113.65 (±11,124.21)	0.055
**COPD**	4835.97 (±8535.16)	5020.58 (±5617.36)	0.255
**Renal insufficiency**	6288.67 (±11,776.8)	8961.8 (±17,227.29)	0.127
**Cardiac renal failure**	5136.56 (±8545.83)	6574.83 (±14,927.49)	0.566
**CCI**			
0	1480.46 (±4646.95)	3538.16 (±6809.92)	**0.011**
1	3214.08 (±4602.83)	5108.24 (±5134.96)	**0.046**
2	4108.37 (±7822.67)	3902.92 (±3645.29)	0.158
3	5399.81 (±13,193.51)	8495.83 (±25,791.21)	0.810
4	4591.78 (±6976.44)	5410.87 (±6587.38)	0.261
5	4936.1 (±8448.64)	5689.64 (±7801.82)	0.160
**Community**			
Andalucía	2289.25 (±3978.57)	NA (±NA)	NA
Barcelona	3236.12 (±7884.45)	2494.36 (±3614.44)	0.891
Galicia	2080.4 (±3809.47)	2685.84 (±3564.29)	0.218
León	925.79 (±1711.17)	NA (±NA)	NA
Madrid	3491.1 (±7313.29)	5528.96 (±10,526.6)	**<0.001**
**Total cost**	3329.17 (±7175.97)	5196.96 (±10,019.56)	**<0.001**

Data are shown as n (%), mean ± sd. Significant comparisons (*p* < 0.05) are marked in bold.

**Table 2:** Estimate and expected number of cases of RSV and total cost before and after vaccine application.

RSV Month	Period	VRS Cases	Reduced Cases Due to Vaccine	Total Cost (€) of Sanitary Uses	Total Cost (€) of Sanitary Uses After Using Vaccine	% of Change
10-2023	1 year	5	0	25,699.26	0.0	−100.0
	2 year		0		0.0	−100.0
11-2023	1 year	46	4	402,720.967	13,991.61	−96.5
	2 year		9		54,543.35	−86.5
12-2023	1 year	77	6	392,023.8	13,991.61	−96.4
	2 year		16		73,575.89	−81.2
01-2024	1 year	70	8	252,768.07	28,528.47	−88.7
	2 year		15		41,001.04	−83.8
02-2024	1 year	10	0	56,062.39	0.0	−100.0
	2 year		2		397.82	−99.3
03-2024	1 year	21	3	60,829.22	596.73	−99.0
	2 year		5		12,702.2	−79.1
Total	1 year	229	21	1,190,103.71	89,448.42	−92.5
	2 year		47		216,320.3	−81.8

**Table 3:** Estimated and expected number of patients and total cost before and after vaccine implementation, segregated by age groups.

Group	RSV Month	Year	VRS Cases	Mean Reduced Cases Due to Vaccine	Total Cost (€) of Sanitary Uses	Reduced Cost (€) of Sanitary Uses Due to Vaccine	% of Change
**60–70 years**	10-2023	1 year	0	0	0.00	0.00	0.0
		2 year		0		0.00	0.0
	11-2023	1 year	14	1	63,096.21	198.91	−99.7
		2 year		3		10,785.34	−82.9
	12-2023	1 year	19	2	69,824.37	9872.99	−85.9
		2 year		4		6351.1	−90.9
	01-2024	1 year	16	2	71,083.06	15,826.27	−77.7
		2 year		4		17,482.78	−75.4
	02-2024	1 year	2	0	4663.87	0.00	−100
		2 year		0		0.00	−100
	03-2024	1 year	4	1	10,270.81	198.91	−98.1
		2 year		1		198.91	−98.1
	Total	1 year	55	6	218,938.32	26,097.08	−88.1
		2 year		12		34,818.13	−84.1
**70–80 years**	10-2023	1 year	2	0	9128.83	0.00	−100
		2 year		0		0.00	−100
	11-2023	1 year	10	0	218,861.127	0.00	−100
		2 year		2		3919.71	−98.2
	12-2023	1 year	23	2	134,817.19	3175.55	−97.6
		2 year		5		26,443.42	−80.4
	01-2024	1 year	11	1	40,980.28	198.91	−99.5
		2 year		2		6896.35	−83.2
	02-2024	1 year	1	0	6697.44	0.00	−100
		2 year		0		0.00	−100
	03-2024	1 year	10	1	34,135.41	198.91	−99.4
		2 year		3		12,304.38	−64.0
	Total	1 year	57	4	444,620.28	3573.37	−99.2
		2 year		12		49,563.86	−88.9
**80–90 years**	10-2023	1 year	0	0	0.00	0.00	0.0
		2 year		0		0.00	0.0
	11-2023	1 year	20	3	111,089.55	13,792.7	−87.6
		2 year		4		39,838.3	−64.1
	12-2023	1 year	26	2	143,078.98	943.07	−99.3
		2 year		5		37,605.82	−73.7
	01-2024	1 year	26	3	75,513.51	9327.74	−87.6
		2 year		5		8038.33	−89.4
	02-2024	1 year	3	0	1886.14	0.00	−100
		2 year		1		198.91	−89.5
	03-2024	1 year	5	1	13,991.61	198.91	−98.6
		2 year		1		198.91	−98.6
	Total	1 year	80	9	345,559.79	24,262.42	−93.0
		2 year		16		85,880.27	−75.1
**+90 years**	10-2023	1 year	3	0	16,570.43	0.00	−100
		2 year		0		0.00	−100
	11-2023	1 year	2	0	9674.08	0.00	−100
		2 year		0		0.00	−100
	12-2023	1 year	9	0	44,303.26	0.00	−100
		2 year		2		3175.55	−92.8
	01-2024	1 year	17	2	65,191.22	3175.55	−95.1
		2 year		4		8583.58	−86.8
	02-2024	1 year	4	0	42,814.94	0.00	−100
		2 year		1		198.91	−99.5
	03-2024	1 year	2	0	2431.39	0.00	−100
		2 year		0		0.00	−100
	Total	1 year	37	2	180,985.32	3175.55	−98.2
		2 year		7		11,958.04	−93.4

Percentage change (% of change) was calculated as (V2-V1)/V1.

**Table 4:** Impact of RSV Vaccine on Healthcare Costs and Case Reductions for Patients with Comorbidities.

Comorbidity	Year	VRS Cases	Reduced Cases Due to Vaccine	Total Cost (€) of Sanitary Uses	Reduced Cost (€) of Sanitary Uses Due to Vaccine	% of Change
Pneumonia	1 year	50	2	303,242.08	22,324.8	−94.5
	2 year		8		36,463.84	−91.0
Bronchitis	1 year	29	2	192,672.72	3720.8	−98.1
	2 year		4		49,858.72	−74.1
Acute infections	1 year	23	1	130,866.76	198.91	−99.8
	2 year		3		12,304.38	−84.8
Renal insufficiency	1 year	62	9	555,631.417	33,192.34	−95.6
	2 year		11		60,496.63	−92.0
Current smoker	1 year	41	3	209,476.9	3374.46	−98.4
	2 year		8		29,073.72	−86.1
Heart failure	1 year	77	6	506,262.167	22,177.37	−95.9
	2 year		11		17,763.89	−96.7
Asthma	1 year	14	1	81,103.48	9674.08	−96.81
	2 year		3		5606.94	−98.2
Neoplasia	1 year	72	7	536,350.357	26,295.99	−88.9
	2 year		15		73,973.71	−68.7
Hypertension	1 year	147	14	751,706.697	28,034.7	−94.5
	2 year		30		104,525.79	−79.4
Diabetes	1 year	47	6	236,486.84	25,898.17	−80.2
	2 year		5		17,482.78	−86.6
COPD	1 year	81	6	406,666.9	10,668.63	−98.1
	2 year		15		68,366.77	−87.7

## Data Availability

The data supporting the reported results cannot be shared due to ethical restrictions.

## Supplementary Materials

The following supporting information can be downloaded at: https://www.mdpi.com/article/10.3390/diseases13020035/s1, Table S1. Breakdown of Healthcare Cost Estimates. Table S2. Results obtained from the Simple Linear Model for Comorbidities Associated with Total Health Costs.
