# Supplementary material for: Burden and Economic Impact of Respiratory Viral Infections in Adults Aged 60 and Older: A Focus on RSV
Source: Diseases. 2025 Jan 28;13(2):35. doi: 10.3390/diseases13020035 (PMC11854486; doi:10.3390/diseases13020035)
Supplement: Supplementary file 1 [file diseases-13-00035-s001.zip › diseases-3405703-supplementary.pdf]

diseases

Table S1. Breakdown of Healthcare Cost Estimates.

Cost Category	Unit Cost (€)
ICU (Intensive Care Unit)	1457.60
Hospital Admission	744.16
Emergency Room	198.91
Oxygen Therapy	8.15
Non-invasive Mechanical Ventilation (VMNI)	249.88
Invasive Mechanical Ventilation (VMI)	312.34

Table S2. Results obtained from the Simple Linear Model for Comorbidities Associated with Total Health Costs.

Variable	Coefficients	Standard Errors	p-values
**Intercept**	5.855	0.051	**<0.001**
**Pneumonia**	1.942	0.098	**<0.001**
**Bronchitis**	1.783	0.143	**<0.001**
**Acute infections**	1.412	0.171	**<0.001**
**Renal insufficiency**	0.438	0.086	**<0.001**
**Current smoker**	0.323	0.107	**0.002**
**Heart failure**	0.297	0.085	**<0.001**
**Asthma**	0.272	0.138	**0.049**
**Neoplasia**	0.227	0.076	**0.003**
**Hypertension**	0.225	0.07	**0.001**
**Diabetes**	0.187	0.085	**0.028**
**COPD**	0.176	0.086	**0.042**
**Alcoholism**	0.105	0.162	0.518
**Former smoker**	0.093	0.082	0.255
**Obesity**	0.051	0.108	0.639

Variables with a significant association (p-value < 0.05) are marked in bold and with an asterisk.
